# Gene-Environment Covariance as a Mechanism of Declines in Openness in Late Adulthood

**DOI:** 10.1093/geroni/igaa057.1495

**Published:** 2020-12-16

**Authors:** Christopher Beam, Emily Sharp

**Affiliations:** 1 Dornsife College of Arts and Letters, Los Angeles, California, United States; 2 Yale University, New Haven, Connecticut, United States

## Abstract

We previously demonstrated that openness to experience declines with age and these declines correlate with mortality risk. We posited that decline in openness was related to change in behavior in relation to a change in future time perspective, defined here as dynamic changes in scope of time that influence persons’ behavior. This idea, based on Baltes’ selection, optimization, and compensation theory of lifespan development, suggests that with foreshortened time horizons individuals adapt their behavior leading to lesser engagement in novel experiences and relationships. The current study examined the genetically informed mechanisms underpinning the relationship between openness and mortality. Using identical and fraternal twins from the Swedish Adoption Twin Study of Aging (SATSA), we examined whether twins further from death nonrandomly select environments that maintain their openness scores while their co-twins nearer to death nonrandomly select environments that contribute to declines in openness. Using a sample of 822 twin pairs, we estimated a genetically-informed longitudinal model that quantified time-varying effects of twins’ openness scores at time t-1 on latent nonshared environmental scores at time t. The model generates within-family gene-environment correlation, a statistical coefficient that quantifies the genetic basis for nonrandom exposure to environments. Results suggest significant time-varying correlations between twins’ openness scores and their unique environments as well as increasing gene-environment correlation over time. Findings are consistent with the view that environments can support and reinforce maintenance of or declines in openness depending on the length of persons’ time horizons.

